# Does women’s place of birth affect their opportunity for an informed choice about Down syndrome screening? A population-based study in France

**DOI:** 10.1186/s12884-021-04041-8

**Published:** 2021-08-30

**Authors:** Olivia Anselem, Marie-Josèphe Saurel-Cubizolles, Babak Khoshnood, Béatrice Blondel, Priscille Sauvegrain, Nathalie Bertille, Elie Azria, for the BiP study group, Olivia Anselem, Elie Azria, Marie-Pierre Bonnet, Marguerite Cognet, Catherine Deneux-Tharaux, Romain Guedj, Morgane Linard, Charlotte Ngo, Juliette Richetin, Anne Rousseau, Marie-Josèphe Saurel, Priscille Sauvegrain

**Affiliations:** 1 Maternité Port-Royal, APHP.Centre-Université de Paris, FHU PREMA, 123 boulevard de Port-Royal, 75014 Paris, France; 2 Université de Paris, CRESS, Obstetrical Perinatal and Pediatric Epidemiology Research Team, EPOPé, INSERM, INRA, Paris, France; 3 Maternité du Groupe Hospitalier Pitié-Salpêtrière, Assistance Publique Hôpitaux de Paris, Université Paris, Paris, France; 4 Maternité du Groupe Hospitalier Paris Saint Joseph, FHU Prema, Paris, France

**Keywords:** Down syndrome, prenatal diagnosis, social disparities, implicit bias

## Abstract

**Background:**

To examine disparities by maternal place of birth in the opportunity to make an informed choice about Down syndrome screening, in France, where the national guidelines recommend that physicians offer it to all pregnant women.

**Methods:**

We used population-based data from the nationally representative French Perinatal Surveys in 2010 and 2016 (*N*=24,644 women) to analyze the opportunity for an informed choice for prenatal screening, measured by a composite indicator.

**Results:**

Among the 24 644 women in the study, 20 612 (83.6%) were born in France, 861 (3.5%) elsewhere in Europe, 1550 (6.3%) in North Africa, and 960 (3.9%) in sub-Saharan Africa. The probability of screening was lower for women born outside France. After adjustment for survey year, maternal age, parity, education level, and the maternity unit’s level of perinatal care, women born outside France had the opportunity to make an informed choice less often than women born in France. This association remained essentially the same even after excluding women without adequate prenatal care.

**Conclusions:**

Women born outside France, including those with adequate prenatal care, had less opportunity than women born in France to make an informed choice about prenatal screening for Down syndrome.

## Background

Down syndrome (trisomy 21) is the most common genetic cause of intellectual disability [1]. Prenatal screening techniques for this disease have advanced considerably in recent decades. In France, the French national authority for health (HAS) coordinates the regularly updated strategy for offering screening in optimal conditions. HAS currently recommends that physicians inform all women that nuchal translucency can be measured at the first ultrasound examination, between 11 weeks and 13 weeks +6 days, and that they can have blood tests. These results enable a risk calculation that integrates the maternal serum markers and can be assayed during the first or second trimester [2]. The methods of Down syndrome screening in France are legally regulated by decrees published successively in 1997, 2009, and 2018 [3], which authorize both physicians and midwives to offer these screening tests. Until 2017, women for whom this screening showed an increased risk of Down syndrome were offered a diagnostic examination — amniocentesis or chorionic villus sampling — to determine fetal karyotype. Only that year did HAS include the study of fetal DNA in its strategy for detecting Down syndrome among women at increased risk [2].

Since 1997, the National Health Insurance Fund had provided full coverage of screening for Down syndrome by the first-trimester ultrasound and the serum marker assays for all women with health insurance. Despite hopes that the disparities in prenatal diagnosis of Down syndrome observed before 1997 would disappear with its full financial coverage, they have persisted, as Khoshnood et al. showed in an analysis of data from the Paris Registry of Congenital Malformations covering the period from 1983 to 2003 [4]. This analysis found progress over time in use of this screening, but also persistence in social disparities, with a screening rate that varied by socioeconomic status and by the mother's place of birth. The proportion of prenatally diagnosed cases of Down syndrome was 15% lower for mothers of African than of French origin. In 2009 a new decree of the HAS recommended the generalization of Down syndrome screening at the first trimester, resulting in an integrated estimate of this risk of Down screening, as it was considered potentially easier to implement than the previous screening strategy. This important change may have affected access to screening for certain social groups, especially foreign-born women, and changed the magnitude of the inequalities previously observed.

Work in other settings has shown similar results with important disparities in screening and in diagnosis. This is the case for example in the Netherlands, where women born in Turkey, North Africa, and the West Indies had rates of screening and diagnosis lower than those of native-born Dutch women [5]. Studies showing disparities in the performance of screening do not, however, allow us to differentiate disparities in access to screening from differences in their use that result from women's deliberate choices. As some authors suggest, social groups may differ in their willingness to undergo an examination so closely related to a strong parental attitude, one that may be culturally or socially determined [6].

In England, Rowe et al. sought to avoid the question of individual choice by focusing on the offer of screening — asking women if the test was offered to them; they observed no geographic inequalities between the more and less advantaged areas [7]. They also found that the test was offered to Asian women less often than to white women. In France, the existence of disparities in access to this screening between women according to their nationality has been demonstrated by analysis of the opportunities they had to make a choice about Down syndrome screening [8]. Although disparities in access fell between 1998 and 2003, in 2003 91% of the women born in France had an opportunity to make a free and informed choice, compared with only 62% of those born in sub-Saharan Africa.

Social inequalities in perinatal health are well documented today, but the reasons for the suboptimal care for certain categories of women remain unclear. Access to health care does not totally explain these discrepancies and further exploration is needed. In particular, unconscious discrimination phenomena may be involved. The existence of implicit bias has been demonstrated among healthcare professionals in several fields of medical practice, but never in the perinatal field. Access to prenatal screening for Down syndrome, where patient information and consent play a fundamental role, is a particularly interesting angle for studying of differential care.

The objective of this study is to analyze the opportunity to make an informed choice about Down syndrome screening and the proportion of women who underwent it in the second decade of this century in a national sample of women in France, according to the mother's place of birth.

## Methods

Data for this analysis come from the National Perinatal Surveys conducted in 2010 and 2016. These cross-sectional surveys collected data from all births at a gestational age of at least 22 weeks or with a birth weight of at least 500 grams during a single week, in all maternity units in metropolitan France and the overseas territories [9].

The data came both from the medical records, mainly on delivery, and the child's condition at birth, and a postpartum face-to-face questionnaire-based interview with parturients before their discharge. This interview focused on their prenatal care and their socioeconomic characteristics [9].

Women who did not speak French, were younger than age 18, or those who experienced a stillbirth or termination of pregnancy were excluded from the study.

In 2010, 13 894 women with 14 142 babies were included, and in 2016, the sample comprised 15 187 women with 15 418 babies. The two survey samples were first compared to verify the absence of major heterogeneity and to analyze the trends in Down syndrome screening uptake. The two samples were then merged and analyzed together to improve the statistical power of the analysis. Merging two survey databases to reach an appropriate size for some groups to allow an adequately powered analysis was possible because 1) the national screening strategies were the same over the two study periods, 2) we were able to test and demonstrate the absence of interaction between survey year and maternal place of birth, and 3) we verified that, although there were differences between the two surveys, these were not major.

We selected women who had lived in metropolitan France for at least one year when they gave birth and whose place of birth was known: 13 507 women in 2010 and 11 137 women in 2016.

### Maternal characteristics studied

The mother's place of birth was classified in five categories: France, other European countries, North Africa, sub-Saharan Africa, and the rest of the world. The regions of the world we chose refer to categories of geographical origin linked to the history of migrations to France. Each category has specific characteristics; women born in other European countries usually have easy access to healthcare in line with the European regulations, while women from North Africa belong to a community integrated in France for several generations with strong social networks, and women from sub-Saharan countries migrated more recently, are more frequently socially isolated, have higher perinatal and maternal risks than the other groups and more frequent social deprivation. The categories France, Europe, North Africa, sub-Saharan Africa, and other countries are often used in most ethnic studies in France and Europe [8].

In both surveys, the questionnaire used as the basis for the face-to-face interview included the following questions about Down syndrome screening: "Did you have fetal nuchal translucency measured at your first-trimester ultrasound to learn your risk of Down syndrome?" and "Did you have a blood test to learn your risk for Down syndrome (serum markers)?" The possible responses were "Yes," "No," or "I don't know". If the answer to the latter question was negative, the reasons could be: refusal; amniocentesis or chorionic villus sampling from the start; tests were not offered; late initiation of prenatal care or no prenatal care or prenatal care abroad; another reason; or "don't know".

**Fig. 1:**
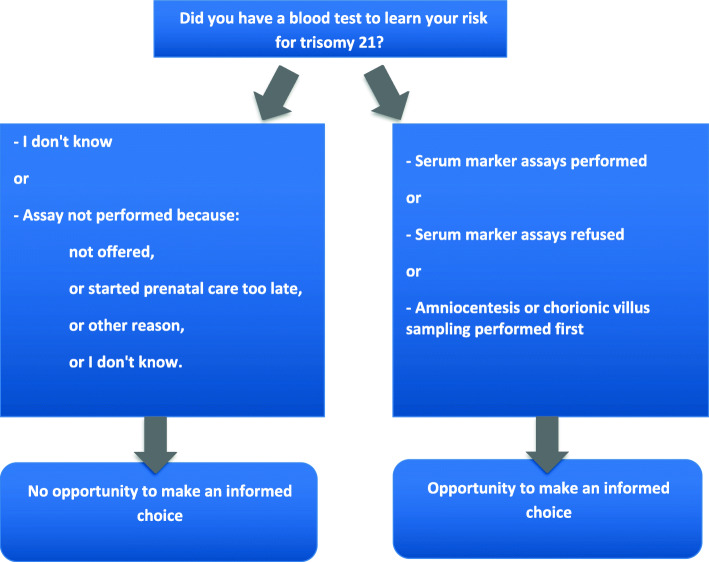
Construction of the indicator: opportunity to make an informed choice about Down syndrome screening

To analyze access to the Down syndrome blood screening test, we used a composite indicator built from these answers defined by Grupposo et al. [8]. Accordingly, we considered that women had an opportunity to make an informed choice about Down syndrome screening if they had undergone or refused a serum marker assay. We also considered that they had an opportunity to make an informed choice if they had an initial amniocentesis or chorionic villus sampling. We considered that women had not had this opportunity when they answered “I don’t know” to the question "Did you have a blood test to learn your risk for Down syndrome (serum markers)?", based on the hypothesis that women who did not remember having this test had not received sufficient information to make an informed choice. We also considered that women did not have the opportunity to make an informed choice if the serum marker assay had not been performed for one of the following reasons: not offered, because of late antenatal care, for another reason, or for an unknown reason (Fig. 1).

Adequate prenatal care was defined in accordance with French clinical practice guidelines [10] by at least 8 antenatal visits and at least 3 prenatal ultrasounds for term births, and according to gestational age at birth for preterm births [9].

The women’s characteristics were previously described in the National Perinatal Survey report [11]. The other covariables considered were age, parity, and educational level.

### Statistical analyses

Pearson's Chi-square tests were used to compare the percentages of women who underwent Down syndrome screening and the opportunity to make an informed choice between 2010 and 2016, according to the mother's country of birth. Differences were considered significant when p was less than 0.05.

The analysis was performed after merging the 2010 and 2016 databases.

To study the association between the mothers' country of birth and proportions of the different prenatal screening tests for Down syndrome, as well as the opportunity to make an informed choice about it, we began by testing the interaction between survey year and maternal birthplace. It did not appear significant. We then used multivariate multinomial logistic regression models adjusted for the survey year, the individual factors (maternal age, parity, educational level, and prenatal care), and for the maternity ward characteristics (level of perinatal care: I, IIA, IIB, and III).

For the analyzed sample, the proportion of missing data per variable ranged only from 0.3% to 0.8% and thus did not justify an analysis with multiple imputations. The results presented are therefore those for the complete cases only.

The statistical analyses were performed with Stata 13 software.

## Results

Among the 24 644 women included, 20 612 (83.6%) were born in France, 861 (3.5%) in another European country, 1550 (6.3%) in North Africa, 960 (3.9%) in sub-Saharan Africa, and 661 (2.7%) in the rest of the world.

Prenatal screening for Down syndrome, whether by nuchal translucency measurements or maternal serum marker assays, rose significantly between 2010 and 2016 (*p*<0.001) (Table 1). The proportion of women reporting that they were not offered serum marker testing dropped from 12.7% in 2010 to 9.5% in 2016. Initial use of amniocentesis or chorionic villus sampling also regressed, from 9.7% in 2010 to 2.2% in 2016. The proportion of women who did not know why their serum markers were not tested fell from 2.6% in 2010 to 2.1% in 2016. On the other hand, refusal to have these markers assayed rose, accounting for 59.1% of the reasons for non-testing in 2016, up from 44.0% in 2010.

**Table 1:** Comparison in Down syndrome screening between 2010 and 2016

	2010 %	2016 %	2010 + 2016 (n) %	p
**Did you have fetal nuchal translucency measured at your first-trimester ultrasound to learn your risk of Down syndrome?**			(24 592)	
No	5.6	5.7	5.6	
Yes	85.1	88.2	86.5	
I don't know	9.3	6.2	7.9	
				<0.001
**Did you have a blood test to learn your risk for Down syndrome (serum markers)?**			(24 484)	
No	12.7	9.5	11.3	
Yes	84.7	88.4	86.4	
I don't know	2.6	2.1	2.4	
				<0.001
**If not, why didn't you have this assay?**			(2 543)	
not offered	14.2	6.8	11.6	
I refused	44.0	59.1	49.2	
prenatal care was: too late/or none/ or abroad	21.4	22.9	21.9	
amniocentesis or chorionic villus sampling	9.7	2.2	7.0	
other reason	5.6	5.2	5.5	
I don't know	5.2	3.8	4.7	
				<0.001
**Opportunity to make an informed choice**^**a**^			(24 265)	
Yes	91.7	94.7	93.0	
No	8.4	5.3	7.0	
				<0.001

^a^ Answer "yes" if the serum marker assay was performed or refused, or if amniocentesis or a chorionic villus sampling was performed first. Answer "no" if the answer to the question "did you have a blood test to learn your risk of Down syndrome (serum markers)?" was "I don't know" or if the assay was not performed for one of the following reasons: not offered, because of late prenatal care, for another reason, or for an unknown reason (see Fig. 1)

The proportion of women who did not have an "opportunity to make an informed choice" was 8.4% in 2010 and 5.4% in 2016 (*p*<0.001).

Cumulatively in the two surveys, women born outside of France underwent Down syndrome screenings less often than women born in France (Table 2): 76.3% of the women born in other European countries, 62.6% of those born in North Africa, and 62.4% born in sub-Saharan Africa reported fetal nuchal measurement, compared with 90.3% of the native Frenchwomen (*p*<0.001). Similarly, 77.9% of the women born in other European countries, 62.7% of those from North Africa, and 69.0% from sub-Saharan African countries reported serum marker assays, compared with 89.5% of the French-born women (*p*<0.001).

**Table 2:** Down syndrome screening according to mother's place of birth (years: 2010+2016)

	Total *n*=24 644 %	France *n*=20 612 %	Europe *n*=861 %	North Africa *n*=1550 %	Sub-Saharan Africa *n*=960 %	Other countries *n*=661 %	p
**Did you have fetal nuchal translucency measured at your first-trimester ultrasound to learn your risk of Down syndrome ?**							
No	5.6	4.6	6.4	13.6	11.5	9.3	
Yes	86.5	90.3	76.3	62.6	62.4	72.3	
I don't know	7.9	5.2	17.3	23.8	26.1	18.4	
							<0.001
**Did you have a blood test to learn your risk for Down syndrome?**							
No	11.3	9.5	14.8	28.0	20.3	11.1	
Yes	86.4	89.5	77.9	62.7	69.0	79.8	
I don't know	2.4	1.1	7.2	9.3	10.7	9.1	
							<0.001
**If not, why didn't you have this blood test?**							
not offered	11.6	11.4	12.5	12.5	12.4	8.3	
I refused	49.2	47.9	50.0	58.3	43.2	46.7	
prenatal care was: too late/or none/or abroad	21.9	22.4	15.2	18.3	27.0	28.3	
amniocentesis or chorionic villus sampling first	7.0	8.8	9.8	1.5	0.60	3.3	
other reason	5.5	6.5	2.7	1.8	6.2	1.7	
I don't know	4.7	2.9	9.8	7.6	10.7	11.7	
							<0.001
**Opportunity to make an informed choice**^**a**^							
Yes	93.0	95.1	87.2	79.9	78.4	86.0	
No	7.0	4.9	12.8	20.1	21.6	14.0	
							<0.001

^a^Answer "yes" if the serum marker assay was performed or refused, or if amniocentesis or a chorionic villus sampling was performed first. Answer "no" if the answer to the question "did you have a blood test to learn your risk of Down syndrome (serum markers)?" was "I don't know" or if the assay was not performed for one of the following reasons: not offered, because of late prenatal care, for another reason, or for an unknown reason (see Fig. 1)

The proportions of women who did not know if they had had nuchal translucency measurements or serum marker assays were significantly higher among the women born abroad than among those born in France.

The analysis showed that the women born outside France had the "opportunity to make an informed choice" less often than the natives. Overall, 12.8% of the women born elsewhere in Europe, 20.1% of those born in North Africa, and 21.6% of those born in sub-Saharan Africa did not have this opportunity, compared with 4.9% of the native Frenchwomen (*p*<0.001).

After adjustment for the survey year, maternal age, parity, educational level, and the maternity ward's level of perinatal care, birth abroad was associated with higher risk of not having the opportunity to make an informed choice (Table 3). Specifically, this was the case for the women born in other European countries (aOR 2.3, 95% CI 1.8-2.9), in North Africa (aOR 3.8, 3.3-4.5) and in sub-Saharan Africa (aOR 3.3, 2.7-3.9).

**Table 3:** Association between mother's place of birth and Down syndrome screening (years: 2010+2016)

	France *n*=20 612 aOR(95% CI)^a^	Europe *n*=861 aOR (95% CI)^a^	North Africa *n*=1550 aOR (95% CI)^a^	Sub-Saharan Africa *n*=960 aOR (95% CI)^a^	Other countries *n*=661 aOR (95% CI)^a^	p
**Did you have fetal nuchal translucency measured at your first-trimester ultrasound to learn your risk of Down syndrome?**						
No	1	1.55 (1.16-2.09)	3.94 (3.30-4.70)	2.58 (2.05-3.24)	3.10 (2.32-4.15)	
Yes	1	1	1	1	1	
I don't know	1	3.36 (2.73-4.13)	5.67 (4.88-6.59)	5.02 (4.20-6.00)	5.57 (4.43-6.99)	
						<0.001
**Did you have a blood test to learn your risk for Down syndrome?**						
No	1	1.67 (1.36-2.05)	3.57 (3.13-4.08)	1.91 (1.60-2.29)	1.31 (1.01-1.70)	
Yes	1	1	1	1	1	
I don't know	1	5.24 (3.81-7.21)	8.30 (6.49-10.62)	6.65 (5.04-8.78)	9.14 (6.58-12.68)	
						<0.001
**Opportunity to make an informed choice**^**b**^						
*All women*	1	2.27 (1.80-2.86)	3.84 (3.28-4.49)	3.27 (2.71-3.94)	3.28 (2.55-4.23)	
						<0.001
*Women who had adequate prenatal care*^***c***^						
	1	2.42 (1.85-3.15)	3.96 (3.31-4.75)	3.20 (2.55-4.00)	3.75 (2.84-4.96)	
						<0.001

^a^ Odds ratio adjusted for survey year, parity, age, mother's educational level and maternity ward level with confidence interval

^b^Answer "yes" if the serum marker assay was performed or refused, or if amniocentesis or a chorionic villus sampling was performed first. Answer "no" if the answer to the question "did you have a blood test to learn your risk of Down syndrome (serum markers)?" was "I don't know" or if the assay was not performed for one of the following reasons: not offered, because of late prenatal care, for another reason, or for an unknown reason (see Fig. 1)

^c^at least 8 prenatal visits and 3 ultrasounds

After exclusion of the women without adequate prenatal care, these risks remained high: ORa 2.4 (1.8-3.1) for the women born in other European countries, ORa 4.0 (3.3-4.7) in North Africa and ORa 3.2 (2.5-4.0) in sub-Saharan Africa.

## Discussion

### Principal results

Our study shows that women born abroad and living in France when they gave birth were less likely to be screened for Down syndrome — either or both of nuchal translucency measurements and serum marker assays — than women born in France. It also shows the proportion of women who did not have the opportunity to choose the serum screening, for which consent is required, was higher for foreign-born women than among women born in France.

Other authors have reported disparities in Down syndrome screening according to women's origin. In the Netherlands, Fransen et al. found a lower rate of participation in Down syndrome screening among women with at least one parent born abroad (North Africa, Turkey, Surinam, West Indies, other Western or non-Western countries) compared with women born to parents both born in the Netherlands [5]. In Australia, Maxwell et al. showed that screening for Down syndrome took place less often for Aboriginal Australian women than for their non-Aboriginal counterparts [12]. Other authors have studied the use of diagnostic examinations such as amniocentesis or chorionic villus sampling and found the same disparities according to women's geographic origin [13, 14].

Nonetheless, these studies cannot differentiate problems in access to these tests from the choice to not undergo screening. That is, the lower frequency of Down syndrome screening among women born abroad may result from their attitude towards this test, determined in part by social or cultural factors [6, 15–17]. Therefore, these studies did not provide information about the ability of the healthcare system to respond to the needs of different groups.

In our study, the use of the composite indicator "opportunity to make an informed choice" makes it possible to study the offer of screening and its disparities by maternal place of birth. Among the women who did not have the opportunity to make a choice, some lacked access to screening because of barriers to it, which we know are numerous among migrant women [18]. Unlike the previous study using this indicator, we also excluded women who arrived in France less than a year before giving birth and women whose prenatal care was considered suboptimal. The hypothesis that these disparities in access to prenatal care determine the disparities in access to an informed choice about this screening, is nonetheless called into question by the nearly identical results we found after those exclusions. These disparities in access to an informed choice are therefore not linked only to the late start of prenatal care. Moreover, because women with problems speaking French were not included, these disparities cannot be attributed only to language barriers either.

Our study thus allows us to show that women born abroad, including those receiving adequate prenatal care, had the opportunity to make an informed choice about Down syndrome screening less often than those born in France; that is, differential care exists in this context. Several hypotheses might explain why some physicians and midwives responsible for telling women about the existence of and the reasons and procedures for these screening tests failed to do so. It is possible that some physicians have prejudices about the attitudes of women born abroad toward this screening or toward terminations of pregnancy after a positive diagnosis. A Delphi consensus process in France thus showed that experts considered that women born in North Africa and Muslim women were less likely to request terminations of pregnancy [19].

These prejudices or implicit biases concerning some ethnic or cultural groups can thus lead to differential care, manifested by the disparities observed here. While the existence of implicit bias has been demonstrated repeatedly by physicians in different specialties [20–26], including in perinatal medicine [27], very little proof exists of its manifestations in terms of differential care [20, 28–30]. Other work nonetheless converges with ours to support this hypothesis. Rowe et al. also distinguished the offer of screening and its performance in England and showed that women born in Asia were offered screening less often [7]. They also showed that when it is proposed, it is chosen less often. Some authors have used scales to study women's attitude toward screening by their socio-occupational category or their geographical origin [17, 31–35]. The prospective study by Dormandy et al. of 1499 women in England showed that women born abroad were no more unfavorable to screening than those born in England; nonetheless the women born abroad knew less about the screening [17].

### Policy implication

Screening for Down syndrome is a complex process, becoming even more complex with technical progress, in particular, the examination of fetal DNA in maternal blood. The level of knowledge of the women who accept it is clearly suboptimal, as demonstrated in France [36]. Our study shows that inadequate information is still more marked among women born abroad, probably because their level of health literacy is inadequate and lower than that of women born in France, but possibly also because some physicians provide differential care related to implicit biases. Other studies are required to explore more precisely the impact of professionals’ implicit bias in this area of healthcare. These works could lead to implementation of educational program with training for health care workers.

### Strengths and limitations of the study

The principal strength of our study is the good quality of the data from representative nationwide populations. Moreover, a face-to-face questionnaire used during an interview within days of delivery asked about the details of the different stages of screening and the reasons why they were not performed. The pooling of data from two different survey years has been taken into account by the statistical analysis.

Moreover, a recall bias cannot be ruled out, to the extent that the information was collected during the postpartum period, but there it could exist equally in all groups. Women who did not speak French well enough to respond to the questionnaire were not questioned and are not included in this analysis. This might create a selection bias attenuating the strength of the association between the mother's place of birth and the availability of an informed choice. Moreover, the composite criterion of "an opportunity to make an informed choice" combines in a single category the women who had screening, those who refused it, and those who had an invasive examination. It is possible that some women had the screening without having understood it or without having been clearly informed about its purpose and its implications, because of language barriers.

### Contribution of the findings to the existing body of knowledge

Our study shows that disparities in Down syndrome screening are not only related to lack of access for foreign-born women, but that other factors are involved that may be related to caregivers’ implicit biases.

## Conclusion

Women born outside France, including those with adequate prenatal care, had less opportunity to make an informed choice about prenatal screening for Down syndrome then those born in France did.

The rapid technical advances in screening for fetal abnormalities have led to increasingly more effective tests, in particular examination of fetal DNA in maternal blood. At the same time, these advances have made the process of prenatal screening for Down syndrome increasingly complex. Hence, there is a greater risk of an increase in disparities in effective and informed access to various prenatal screening techniques. One result of these processes is an increase in the probability and degree of implicit bias in the provision of screening tests.

## Data Availability

The data supporting the conclusions of this article are available in the ADISP website https://clicktime.symantec.com/3LxK1SeTZznFcNij7TwrWhE6H2?u=www.progedo-adisp.fr%2Fenquetes_sante.php

## Abbreviation

HAS: Haute Autorité de Santé French national authority for health
